# Identification of Highly Sensitive Pleural Effusion Protein Biomarkers for Malignant Pleural Mesothelioma by Affinity-Based Quantitative Proteomics

**DOI:** 10.3390/cancers15030641

**Published:** 2023-01-19

**Authors:** Nicolai B. Palstrøm, Martin Overgaard, Peter Licht, Hans C. Beck

**Affiliations:** 1 Department of Clinical Biochemistry, Odense University Hospital, 5000 Odense, Denmark; 2 Department of Clinical Research, University of Southern Denmark, 5000 Odense, Denmark; 3 Department of Cardiothoracic and Vascular Surgery, Odense University Hospital, 5000 Odense, Denmark

**Keywords:** proteomics, malignant mesothelioma, fibulin-3, mesothelin, pleural effusion biomarkers

## Abstract

**Simple Summary:**

The development of malignant pleural mesothelioma, a rare and often aggressive cancer associated with asbestos exposure, can take decades to develop. The existing methods for diagnosis are insufficient, hence, better detection methods are required. Given that pleural effusions are close to the tumor and reasonably accessible, it is believed that pleural effusion contains biomarkers that can provide insight into the disease. As part of our explorative analysis of pleural effusion, we applied a novel mass spectrometry-based method, and as a result, we have identified several proteins with diagnostic potential as markers for malignant pleural mesothelioma. The research into the potential use of pleural effusion biomarkers as a viable future diagnostic tool for malignant pleural mesothelioma will be advanced with the addition of the knowledge gained from our study.

**Abstract:**

Malignant pleural mesothelioma (MPM) is an asbestos-associated, highly aggressive cancer characterized by late-stage diagnosis and poor prognosis. Gold standards for diagnosis are pleural biopsy and cytology of pleural effusion (PE), both of which are limited by low sensitivity and markedly inter-observer variations. Therefore, the assessment of PE biomarkers is considered a viable and objective diagnostic tool for MPM diagnosis. We applied a novel affinity-enrichment mass spectrometry-based proteomics method for explorative analysis of pleural effusions from a prospective cohort of 84 patients referred for thoracoscopy due to clinical suspicion of MPM. Protein biomarkers with a high capability to discriminate MPM from non-MPM patients were identified, and a Random Forest algorithm was applied for building classification models. Immunohistology of pleural biopsies confirmed MPM in 40 patients and ruled out MPM in 44 patients. Proteomic analysis of pleural effusions identified panels of proteins with excellent diagnostic properties (90–100% sensitivities, 89–98% specificities, and AUC 0.97–0.99) depending on the specific protein combination. Diagnostic proteins associated with cancer growth included galactin-3 binding protein, testican-2, haptoglobin, Beta ig-h3, and protein AMBP. Moreover, we also confirmed previously reported diagnostic accuracies of the MPM markers fibulin-3 and mesothelin measured by two complementary mass spectrometry-based methods. In conclusion, a novel affinity-enrichment mass spectrometry-based proteomics identified panels of proteins in pleural effusion with extraordinary diagnostic accuracies, which are described here for the first time as biomarkers for MPM.

## 1. Introduction

Malignant mesothelioma (MPM) is a rare and aggressive type of cancer arising from mesothelial cells of the pleural, pericardial, and peritoneal cavities. Asbestos inhalation is the predominant cause of MPM, with approximately 80% of patients having been exposed to asbestos, either directly through occupation or through a family member [1]. Asbestos has been used as a heat insulator and fireproofing material in construction, and as such, there still exists housing today with asbestos as an insulating material. Inhaled fibers promote carcinogenesis via the induction of inflammatory reactions and subsequent immune responses [2]. Current treatment options include chemotherapy, surgery, and radiotherapy, or a combination of the three [3]. The prognosis is poor, with a 5-year survival rate below 15%, and median survival is approximately 12 months from diagnosis [4]. It has been proposed that early diagnosis would improve the treatment options and outcome of MPM. However, because of the notorious difficulty in diagnosing these tumors, research in this field remains focused on the identification of biomarkers in pleural effusions and other biological fluids that could serve as a diagnostic tool for identifying mesothelioma. Pleural effusion is thought to be apreferable source for initial biomarker discovery because it is a body fluid that is tumor-proximal with a relatively straightforward extraction opportunity using pleuracentesis. Potential biomarkers are currently investigated for their use to predict, diagnose and monitor but none has yet proved useful. Several proteins have been proposed as molecular biomarkers for MPM; among those who have been investigated most extensively are mesothelin and fibulin-3. Mesothelin is a glycoprotein that is expressed on the surface of both benign and malignant mesothelial cells but is overexpressed in MPM and other cancers and can shed from the surface and circulate. Soluble mesothelin have been measured in both pleural effusion and serum, but the low sensitivity has so far prevented its widespread use as screening tool [5,6]. The secreted glycoprotein fibulin-3 has been shown to play a role in the regulation of cell proliferation and migration. Furthermore, levels of fibulin-3 have been found to be elevated in both plasma and pleural effusions in MPM patients compared to non-MPM patients [7]. Meta-analysis has shown fibulin-3 to have moderate diagnostic efficacy [8]. 

It is well-accepted that disease progression affects the expression of specific proteins in diseased tissue, e.g., in various cancers, thus leading to disease-specific protein profiles detectable in tissues and body fluids. Mass spectrometry-based proteomics offers measurement of a global protein profile, which through comparisons of protein expression between benign and malignant samples, can be used as a tool for biomarker discovery in clinical research. This technology has previously proven to be excellent at the identification of promising protein biomarkers in pleura from MPM patients [9]. In this study, we hypothesized that pleural effusions from patients diagnosed with mesothelioma contain yet unreported protein biomarkers that can be identified using a novel affinity-enrichment mass spectrometry-based proteomics method and machine learning algorithms. In this study, we aimed to use this analytical strategy to identify novel protein biomarkers for MPM.

## 2. Materials and Methods

### 2.1. Patients and Patient Samples

Following informed consent, pleural effusions were collected for later proteomic analysis in patients referred for thoracoscopy due to a clinical suspicion of MPM. All patients that were scheduled for a thoracoscopic biopsy to rule out malignant mesothelioma have a history of asbestos exposure during the past decades. Approximately 20 mL of the pleural effusion was collected in the period from September 2012 to November 2016 during thoracoscopy from each patient and immediately centrifuged (2000× *g*/10 min/room temperature) before storage of the supernatant at −80 °C until use. Pleural biopsies was subjected to routine immuno-histological staining for the following markers; calretinin, CDX2, CEA-M, CK7, D2-40, EMA, GATA-3, HBME-1, PAX8, PSA, TTF-1, WT1, and vimentin. Immuno-histological staining of biopsies in conjunction with microscopic observations was used to diagnose malignant mesothelioma.

### 2.2. Sample Processing

For the analysis of high abundant pleural proteins, pleural effusion samples (10 µL) were diluted with 90 µL PBS to which 50 mM dithiothreitol (DTT) (30 min at 50 °C) was added. Next, 150 mM iodoacetamide (IAA) (30 min at RT) was added, followed by enzymatic digestion of proteins with 2 µg of trypsin overnight at 37 °C.

For the analysis of low abundant proteins, their enrichment was carried out by adding 20 µL ABA-affinity beads slurry to 1 mL of diluted pleural effusion (100 µL pleural effusion, 900 µL PBS) and incubated for two hours at RT virtually as previously described [10]. Beads with bound proteins were washed six times with 400 µL PBS and then re-dissolved in 20 µL, 0.2 M TEAB. Affinity-enriched proteins were on-bead reduced, alkylated, and digested as described above by using 0.25 µg trypsin instead of 2.0 µg.

### 2.3. Isobaric Labelling of Tryptic Peptides

Digested peptides were chemically labeled with 11-plex tandem mass tags (TMT; Thermo Fisher Scientific, Rockford, IL, USA) according to vendor instructions. Briefly, five µg of tryptic digest from each sample was randomly labeled with either of the TMT tags 127N, 127C, 128N, 128C, 129N, 129C, 130N, 130C, 131N, and 131C and mixed in equal ratios with a peptide pool of all samples tagged with mass tag 126. Ion signals from this mass tag was used for data normalization across TMT datasets and for the calculation of relative protein abundances.

### 2.4. Proteome Analysis

#### 2.4.1. Off-Line Fractionation

The resulting TMT sets were fractionated into 6 fractions by high-pH fractionation virtually, as previously described [11]. Briefly, samples were loaded onto an ACQUITY UPLC^®^ M-Class CSHTM C18 column (130 Å, 1.7 µm bead size, 300 µm id × 100 mm length) using a 25 min linear gradient from 10% solvent B (20 mM ammonium formate in 80% acetonitrile (ACN), pH 9.3) to 55% solvent B at 6 µL/min flowrate on a Dionex Ultimate 3000 RSLnano system inline coupled to a Dionex 3000 Ultimate UV detector (210 nm) and a Dionex Ultimate 3000 autosampler configured as a fraction collector (Thermo Scientific, Bremen, Germany).

#### 2.4.2. Orbitrap Mass Spectrometry

Nano-LC-MS/MS analysis of higher abundant pleural proteins was performed on an Orbitrap Exploris 480 mass spectrometer (Thermo Fisher Scientific) equipped with an EASYspray source coupled to a Dionex UltiMate 3000 nano-HPLC. Peptide samples (5 µL) were loaded onto an Acclaim PepMap 100 C18 precolumn with a flow of 15 µL per minute for 20 min. Trapped peptides were separated on an EASY spray column at 45 ºC using a linear gradient from 95–76% A (0.1% formic acid) to 25–27.5% B (0.1% formic acid in 100% acetonitrile) over an effective gradient of 104 min at a flowrate of 300 nl per minute. MS1 spectra were acquired at a resolution of 120 K at 200 m/z with a normalized AGC of 100% and peptides were dynamically excluded for 30 s. MS2 spectra were acquired at 45 K resolution at 200 m/z with a normalized AGC target of 100% and normalized collision energy (NCE) set to 36% with a cycle time of 2 s and 1.2 m/z isolation window.

Nano-LC-MS/MS analysis of lower abundant pleural proteins isolated by affinity-enrichment was analyzed on an Orbitrap Eclipse Tribrid mass spectrometer (Thermo Fisher Scientific, San Jose, CA, USA) coupled to a Dionex UltiMate 3000 nano-HPLC. Purified peptides (5 µL) were loaded onto an in-house packed C18 pre-column (2 cm length, OD 360 µm, 75 µm ID packed with ReproSil-Pur C18, 3 µm resin (Dr. Maisch HPLC GmbH, Ammerbuch-Entringen. Germany)) with a flow of 3.5 µL/min for 9 min. Peptides were separated on a column (25 cm, 75 μm ID, in-house packed with ReproSil-Pur C18, 1.9 μm resin (Dr. Maisch GmbH)) using a linear gradient from 95%A to 28%B over 49 min at a flow rate of 250 nL per minute, followed by 8 min at 90%B and 5 min at 98%A at a flow rate of 300 nL per minute. MS1 spectra were acquired at a resolution of 120 K at 200 m/z with a normalized AGC of 100% and peptides were dynamically excluded for 30 s. MS2 spectra were acquired at 50 K resolution at 200 m/z with a normalized AGC target of 250% and NCE set to 40% with a cycle time of 3 s and 0.7 m/z isolation window.

#### 2.4.3. Targeted Analysis of Mesothelin and Fibulin-3 by Multiple-Reaction-Monitoring Mass Spectrometry

Pleural effusion samples for targeted analysis were essentially prepared as previously described [12], but with sample handling in 96-well plates and the use of a premix of trifluoroethanol/DTT. Pre-specified endogenous peptides belonging to mesothelin (IQSFLGGAPTEDLK) and fibulin-3 (DIDECDIVPDACK and NPCQDPYILTPENR) were individually adjusted to a 1:1 ratio with the corresponding spiked-in heavy isotope-labeled standard peptides (SpikeTides_L, JPT Peptide technologies, Berlin, Germany). Peptide samples (0.5 µg) were loaded onto Evotips according to the manufacturer instructions and analyzed using an Evosep One HPLC (8 cm analytical column, 60 samples/day LC method) coupled to a TSQ Altis triple quadrupole mass spectrometer, equipped with an EASY-spray ion source (Evosep Biosystems, Odense, Denmark, and ThermoFisher Scientific GmbH, Bremen, Germany).

#### 2.4.4. Raw Data Processing

All raw data files were processed using the Proteome Discoverer software (v. 2.4.0.305) and searched with the MSPepSearch and the Sequest HT search algorithm. The search parameters for the MSPepSearch were kept at default except for the precursor tolerance and the fragment tolerance were both set to 15 ppm. The TMT specific spectral library was prepared by Shen et al. [13] and imported into Proteome Discoverer. Sequest HT search parameters were set to default except for MS accuracy of 8 ppm, MSMS accuracy of 0.05 Da for HCD data, with two missed cleavages allowed. Fixed modifications were set to carbamidomethylation at cysteine residues, TMT 6-plex N-terminal, and TMT 6-plex on lysine residues. Variable modifications were set to methionine oxidation, deamidation of asparagine and glutamine and N-terminal acetylation. Raw data files were searched against the Swiss-Prot database restricted to the human proteome (downloaded on 12 December 2019, containing 20,303 entries). Proteins identified with at least one unique peptide and with a high confidence (FDR < 1%) were permitted in the final dataset. MRM MS raw files were processed in Skyline (version 21.1.0.278) [14]. Each raw peptide L/H ratio was normalized based on the average of all L/H ratios of that peptide across samples. Protein levels were then normalized to the mean of the reference group.

### 2.5. Statistical Analysis

Imputation of missing values was performed by applying an imputation method based on k-Nearest Neighbors algorithms as previously described [15,16]. Feature selection was performed to identify proteins with the capability to differentiate between disease states and to remove noisy features prior to classifier training. Feature selection was performed using the Boruta feature selection algorithm [DOI: 10.18637/jss.v036.i11]. An additional filter was employed as the Boruta selected proteins was required to also be significantly upregulated (*p* < 0.05, unadjusted) in MPM samples when compared to controls as determined by Student’s *t*-test. Recursive feature elimination was performed to further refine the proteins used for training the classifiers. The final list of proteins for each dataset was then used to train classifiers. Subtype-specific proteins used for classifier training for discriminating between biphasic and epithelioid subtypes were identified by Student’s *t*-test. Development of a classification model for the detection of mesothelioma in pleura was performed in R (v. 4.02) and facilitated by the Random Forest algorithm (RandomForest and caret packages), which is a decision tree-based machine-learning algorithm, using leave-one-out cross-validation for an accurate estimate of performance. Computation of sensitivity, specificity, positive prediction value (PPV), negative prediction value (NPV), as well as diagnostic accuracy, was achieved using the confusionMatrix function in the caret R package. ROC curves and AUC were computed using the MLeval package.

## 3. Results

A total of 84 pleural effusion samples were available for analysis with mass spectrometry. Immunohistology of pleural biopsies confirmed MPM in 40 patients (Table 1) and ruled out MPM in 44 patients who served as controls. The samples were prepared for proteome analysis using two complementary sample preparation methods; a standard sample preparation method that enables the analysis of the most abundant proteins in PE, and a complementary affinity-enrichment-based method that targets low abundant proteins in plasma and other body fluids with protein compositions comparable with plasma [10,17]. The two sample sets were analyzed by mass spectrometry-based proteomics resulting in two datasets with primarily high or low-abundant PE proteins. Each of the datasets were subsequently analyzed with statistical analysis to identify biomarkers and panels of biomarkers for MPM. Finally, two PE proteins previously regarded as potential MPM biomarkers were analyzed by two complementary mass spectrometry methods to validate our proteomics methods.

### 3.1. Identification of Biomarkers for Malignant Mesothelioma

We analyzed a total of 84 pleural effusion samples from 40 MPM patients and 44 patients with benign effusions by mass spectrometry-based proteomics. Statistical analysis of both datasets identified a total of 16 protein biomarker candidates for MPM (Table 2). Ideally, for measuring purposes, diagnostic and prognostic biomarkers should be upregulated when reflecting a disease state. Therefore, only proteins that were significantly upregulated in MPM samples were considered in the present study.

**Figure 1:**
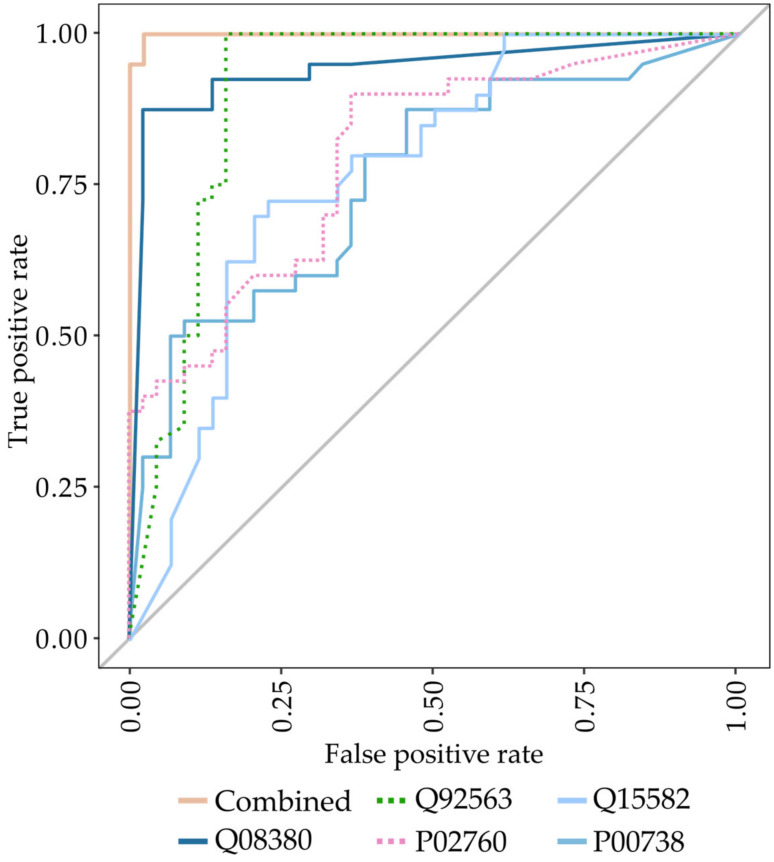
ROC analysis of the combined 5-protein classifier with individual curves for each constituent protein. Q08390: galectin-3-binding protein; Q92563: testican-2; P02760: protein AMBP; Q15582: Beta ig-h3; P00738: haptoglobin.

Next, we analyzed the proteins shown in Table 2 using a Random Forest supervised machine learning algorithm to identify proteins or combinations hereof with capabilities of discriminating MPM patients from non-MPM individuals. The groups of proteins identified from the affinity-based enrichment experiment and from the standard experiment were analyzed both separately and in combination. For the standard proteomics experiment, a single 3-protein Random Forest classifier was identified, whereas the affinity-based proteomics experiment identified a 4-protein classifier (Table 3). Each of the two classifiers demonstrated a high degree of sensitivity (0.90–0.97) and specificity (0.89–0.93), with the classifiers trained on the proteins identified from the standard experiment slightly outperforming the classifier trained on the proteins obtained from the affinity-enriched dataset (accuracy: 0.95 vs. 0.89). The diagnostic power of the identified biomarker panels was further evaluated by receiver-operating characteristic (ROC) analysis for MPM versus non-MPM (Table 3). The area under curve (AUC) values confirmed that both classifiers could discriminate MPM patients from non-MPM patients with the classifiers trained on enriched data achieving an AUC of 0.97. When combining the list of proteins identified from the standard experiment and the affinity-enrichment experiment, a 5-protein classifier could be constructed that demonstrated extreme performance with a sensitivity of 1.00, specificity of 0.98, and an AUC of 0.99 (Figure 1).

### 3.2. Analysis of the Previously Identified MPM Protein Biomarkers Fibulin-3 and Mesothelin

**Figure 2:**
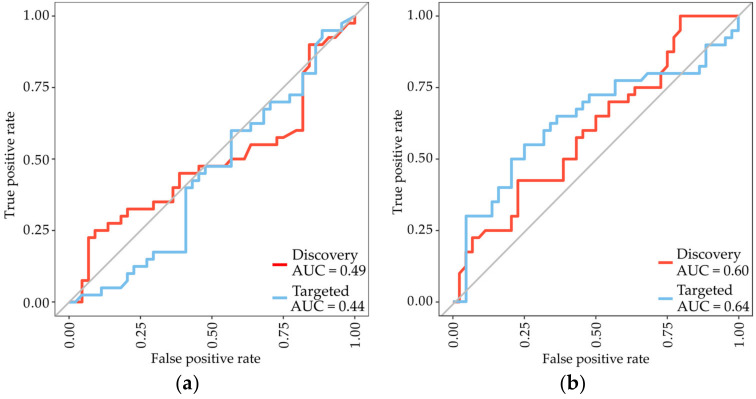
ROC analysis of Random Forest classifiers trained on either (**a**) mesothelin or (**b**) fibulin-3, measured with either discovery-based MS (red) or a confirmative targeted MS assay (blue).

Other proteins present in our datasets include fibulin-3 and mesothelin. Although both proteins were in numerous previous studies identified as biomarkers for MPM [7,8,18,19], none of them were identified as potential MPM biomarkers in the present study. In fact, ROC analysis for both proteins showed similar poor performance with an AUC of 0.49 (95% CI: 0.38–0.64) for mesothelin and 0.60 (95% CI: 0.48–0.72), respectively, for fibulin-3 when measured with discovery-based MS. To further validate these findings we used a confirmative targeted MS assay and obtained comparable results, adding further validity to our study data (Figure 2).

### 3.3. Identification of Subtype-Specific Proteins for Differentiating MPM Subtypes

**Figure 3:**
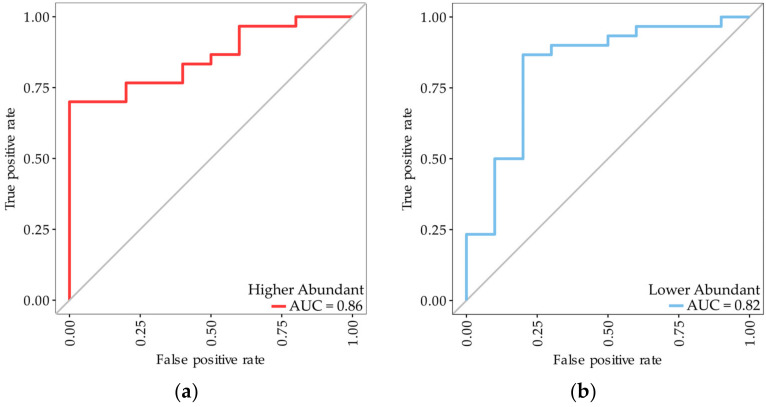
ROC analysis of Random Forest classifiers trained on either the (**a**) higher abundant or (**b**) lower abundant proteins from Table 4, for classification of MPM subtypes. The epithelioid subtype was selected as positive for the calculation of sensitivity and specificity.

We then sought to examine whether the performance of the classifiers were associated with a particular subtype. It was determined using the Student’s *t*-test that there were no significant differences in protein expression between the biphasic and epithelioid subtype for the proteins included in the classifiers trained in either the standard dataset (*p*-value: 0.696–0.527) or the affinity-enriched dataset (*p*-value: 0.978–0.107). This indicates that the performance of the classifiers were not dependent on any particular subtype, but rather, the presence of mesothelioma itself. To further explore the relevance of subtypes we identified the five most significantly regulated proteins between biphasic and epithelioid subtypes using Student’s *t*-test in both datasets (Table 4). These proteins were then used to train two Random Forest classification models to distinguish between the biphasic and epithelioid subtypes. The two classifiers demonstrated a high degree of sensitivity (0.83–0.90) but lower specificity (0.50–0.60), with the classifier trained on the proteins identified from the affinity-enriched dataset, being slightly more accurate than the classifier trained on proteins from the standard experiment (accuracy: 0.78 vs. 0.80). The AUC values retrieved from the ROC analysis indicate that both classifiers could discriminate between patients with different subtypes with the classifier trained on proteins from the standard proteins (AUC (95% CI) = 0.86 (0.74–0.97) slightly outperforming the classifier trained on proteins from the enriched dataset (AUC (95% CI) = 0.82 (0.65–0.99) (Figure 3).

## 4. Discussion

Our experimental proteomics approach relied on the relative quantification using isobaric tags (11-plex tandem mass tags) high-pH fractionation and nano-LC-MSMS analysis followed by bioinformatics and biostatistical analysis. The pleural effusions were prepared using two different methods prior to the proteomic analysis: in one method, samples were prepared for proteomic analysis directly without any pretreatment, which allows measurement of primarily the most abundant proteins present, and in the other we used a novel protein enrichment method [10,17] that enables the detection of proteins, which are present in very low concentrations in the pleural effusion. Using this experimental approach, we measured not only the previously identified and relative abundant protein biomarkers for MPM, fibulin-3, and mesothelin, but also several proteins that have not previously been identified as protein biomarkers for MPM with high diagnostic accuracy when used in combination (Table 3). These included a multimarker panel consisting of galectin-3-binding protein, periostin, and haptoglobin identified among the high abundant proteins (AUC = 0.99), and one multimarker panel identified using our novel affinity enrichment-based proteomics method consisting of testican-2 and nipped-B-like protein combined and with Beta ig-h3 and protein AMBP (Table 3), demonstrating an AUC of 0.97 and sensitivity and specificity of 0.90 and 0.89. When combining three of the proteins from the latter panel with galectin-3-binding protein and haptoglobin identified with the standard proteomics experiment a 5-protein multimarker panel exhibiting an extreme diagnostic accuracy was obtained (AUC = 0.99; sensitivity = 1.00, specificity = 0.98). All the identified multimarker panels for MPM demonstrated high diagnostic performance. This is not surprising as several of the identified proteins previously are associated with various cancers, including MPM (Table 5). For example, the increased expression of nipped-B-like protein is significantly linked with poor prognosis, tumor differentiation, and lymph node metastasis in non-small cell lung cancer [20], Beta ig-h3 plays a role in mesothelioma tumorigenesis and progression [21], periostin, haptoglobin, protein AMBP fragments are upregulated in various cancers including MPM [22,23,24], whereas galectin-3 binding protein are associated to prognosis and progression of various cancers [25].

Present in our dataset were also the proteins fibulin-3 and mesothelin—both originally proposed as biomarkers for MPM [7]. Fibulin-3, a secreted glycoprotein that promotes tumor growth and invasion [18]—was first proposed as a possible MPM pleural biomarker by Pass et al. [7] with excellent capability to discriminate MM patients from non-MM patients (AUC = 0.99; sensitivity = 0.97, specificity = 0.95). This could not, however, be confirmed in a subsequent study by Kirschner et al. [19], who found—in line with our results—that fibulin-3 levels in pleural effusions were not significantly different between MM patients and controls, but demonstrated that fibulin-3 levels in pleural effusion fluid were independently associated with prognosis (hazard ratio of 9.92 (95% CI: 2.14–45.93)), also in line with the results obtained by Battolla et al. [31]. Mesothelin—a 40 kDA cell surface glycoprotein expressed by mesothelial cells—another putative pleural effusion biomarker for mesothelioma—displayed a similar diagnostic effectiveness in our study with an AUC of 0.49 (95% CI: 0.38–0.64), which was lower than the previously reported AUCs ranging from 0.70 to 0.93 [32]. Other newly discovered pleural MPM biomarkers include cytokeratine-19 fragment (CYFRA-21-1) and carcinoembryonic antigen (CEA) [32]. CYFRA-21-1 is the soluble fragment of keratin 19 and is released into circulation after caspase-3 cleavage or carcinogenesis-related apoptosis and has demonstrated AUC values from 0.65 to 0.76 [33,34]. Carcinoembryonic antigen (a glycoprotein involved in cell adhesion) is found in low levels in healthy individuals and in high levels in different cancers and also in MPM, but only in some studies, indicating that this protein is not specific to MPM (AUCs ranging from 0.2 to 0.94) [33,35], thus making this less useful as a differential diagnostic marker for MPM. Improved performances were observed when combining pleural biomarkers. For example, the combination of C–C motif chemokine ligand 2, galectine-3, and secretory leukocyte protease inhibitor with soluble mesothelin-related peptides really improved MPM diagnosis (AUC 0.968) [36]. Moreover, when combining mesothelin with the cytokeratin 21-1/carcinoembryonic antigen-ratio, a sensitivity of 93.4% and specificity of 64.9% was obtained [37]. Furthermore, when combining carbohydrate antigen 15-3 (CA 15-3) with CYFRA 21-1, a sensitivity of 100% and a specificity of 83% were obtained [36].

Our study has limitations: we did not take into account the impact of the different MPM subtypes on the feature selection of biomarkers. It is likely that some of the candidate proteins would have a greater ability to classify patients with a particular subtype. Investigation into this potential discrepancy would have been possible if additional samples from each subtype were available. On the other hand, the analysis of the well-known MPM biomarkers fibulin-3 and mesothelin by a complementary and more sensitive mass spectrometry method confirmed previously reported data that, in all, adds validity to our results. Additional patient data beyond MPM histology, age, and sex was not available for this study preventing the division of patients by asbestos exposure and tumor staging.

## 5. Conclusions

In this study, we identified novel biomarkers for MPM in pleural effusions using mass spectrometry-based proteomics. We demonstrated that the application of our mass spectrometry-based approach, together with machine learning, can assist with the identification of potentially clinically useful biomarkers for the detection of MPM. Future studies should validate these findings in a separate cohort of patients and investigate the possible impact of MPM subtypes on biomarker selection, as well as the implementation of machine learning in the mass spectrometry-based diagnosis of MPM.

## Figures and Tables

**Table 1:** Patient characteristics.

Variables	MPM (n = 40)	Benign (n = 44)
Age (mean ± SD)	71.54 ± 8.61	68.88 ± 8.82
Gender		
Male (%)	35 (87.5)	36 (81.8)
Female (%)	5 (12.5)	8 (18.2)
MM Histology (%)		
Biphasic	10 (25)	-
Epithelioid	26 (65)	-
Unknown	4 (10)	-
Benign Histology (%)		
Fibrosis		8 (18)
Inflammation		11 (25)
Nonspecific reactive change		19 (43)
Others *		6 (14)

* Others include hyperplasia, sclerosis, degenerative changes, and focal atypia.

**Table 2:** Proteins with the capability to discriminate MPM pleural effusions from controls identified by the Boruta feature selection algorithm as described in Section 2.5. Galactin-3-binding protein was identified in both datasets whereas periostin, haptoglobin, vitamin D-binding protein, and fibulin-3, were identified in the dataset comprising of high-abundant proteins. The remaining proteins were only identified in the dataset with the low-abundant proteins.

SwissProt ID	Protein Name	Gene Name	Fold-Change	*p*-Value	Importance Score
Q08380	Galectin-3-binding protein	LGALS3BP	1.51 ^#^/1.69 *	1.44 × 10^−5 #^/8.22 × 10^−12^ *	10.31 ^#^/29.27 *
Q15063	Periostin	POSTN	1.31	0.018	8.09
P00738	Haptoglobin	HP	1.22	0.0011	6.90
P02774	Vitamin D-binding protein	GC	1.08	0.0036	6.54
Q12805	Fibulin-3	EFEMP1	1.12	0.004	6.46
Q92563	Testican-2	SPOCK2	2.78	1.33 × 10^−11^	23.67
Q15582	Beta ig-h3	TGFBI	1.56	6.44 × 10^−7^	15.16
P02760	Protein AMBP	AMBP	1.35	5.28 × 10^−10^	12.79
Q6KC79	Nipped-B-like protein	NIPBL	1.64	0.00037	7.26
P21741	Midkine	MDK	1.63	0.0013	7.21
Q96GQ7	DEAD box protein 27	DDX27	1.40	0.01	6.94
Q6ZRQ5	Protein MMS22-like	MMS22L	1.80	0.0015	6.94
Q99715	Collagen alpha-1(XII) chain	COL12A1	1.42	0.00290	6.31
Q8WWA0	Intelectin-1	ITLN1	2.26	0.0049	5.70
O00468-6	Agrin	AGRN	1.56	0.00051	5.21

Determined in the * lower abundant dataset and the ^#^ higher abundant dataset.

**Table 3:** Diagnostic accuracy of biomarker panels of proteins identified by Random Forest supervised machine learning algorithm. Classifiers were trained using the proteins identified in Table 2. Proteins from the two datasets were analyzed separately and in combination. The proteins analyzed were: Q08380: galectin-3-binding protein; P00738: haptoglobin; Q15063: periostin. Q92563: testican-2; Q6KC79: nipped-B-like protein; Q15582: Beta ig-h3; P02760: protein AMBP.

Variables	Sensitivity	Specificity	PPV	NPV	Accuracy	AUC (95% CI)
**Higher abundant proteins** Protein accession (gene name) Q08380 (LGALS3BP), P00738 (HP), Q15063 (POSTN)	0.97	0.93	0.93	0.97	0.95	0.99 (0.98–1.00)
**Lower abundant proteins** Q92563 (SPOCK2), Q6KC79 (NIPBL), Q15582 (TGFBI), P02760 (AMBP)	0.90	0.89	0.88	0.91	0.89	0.97 (0.94–1.00)
**Combined** Q92563 (SPOCK2), P00738 (HP), Q15582 (TGFBI), P02760 (AMBP), Q08380 (LGALS3BP)	1.00	0.98	0.98	1.00	0.99	0.99 (0.99–1.00)

**Table 4:** Five most significantly regulated proteins between biphasic and epithelioid MPM subtypes in the two datasets.

SwissProt ID	Protein Name	Gene	Fold-Change (Biphasic/Epithelioid)	*p*-Value
Higher abundant proteins				
Q9HC84	Mucin-5B	MUC5B	1.49	0.00068
P23381	Tryptophan-tRNA ligase	WARS1	1.42	0.0023
Q15582	Beta ig-h3	TGFBI	1.31	0.0025
P04217	Alpha-1B-glycoprotein	A1BG	1.10	0.0043
Q9Y240	C-type lectin domain F11A	CLEC11A	1.62	0.0054
Lower abundant proteins				
Q4ZHG4	Fibronectin type III protein 1	FNDC1	2.23	2.47 × 10^−5^
P13611	Versican core protein	VCAN	2.12	0.00064
P12107	Collagen alpha-1(XI) chain	COL11A1	1.83	0.00075
P35442	Thrombospondin-2	THBS2	1.67	0.0017
Q16363	Laminin subunit alpha-4	LAMA4	1.62	0.0018

**Table 5:** Proteins included in the classifiers for either MPM or subtype classification. Proteins are annotated with the associated gene ontology biological process and whether they have previously been shown to be related to either mesothelioma or other cancer forms.

SwissProt ID	Protein ID	Gene	Cancer-Related	Biological Process
Q08380	Galectin-3-binding protein	LGALS3BP	Yes [25]	cell adhesion
Q15063	Periostin	POSTN	Yes [22]	cell adhesion
P00738	Haptoglobin	HP	Yes [23]	acute-phase response
Q92563	Testican-2	SPOCK2	No	extracellular matrix organization
Q15582	Beta ig-h3	TGFBI	Yes [21]	angiogenesis
P02760	Protein AMBP	AMBP	Yes [24]	cell adhesion
Q6KC79	Nipped-B-like protein	NIPBL	Yes [20]	cellular response to DNA damage
Q9HC84	Mucin-5B	MUC5B	No	
P23381	Tryptophan--tRNA ligase	WARS1	No	angiogenesis
P04217	Alpha-1B-glycoprotein	A1BG	No	
Q9Y240	C-type lectin domain F11A	CLEC11A	Yes [26]	ossification
Q4ZHG4	Fibronectin type III protein 1	FNDC1	Yes [27]	
P13611	Versican core protein	VCAN	Yes [28]	cell adhesion
P12107	Collagen alpha-1(XI) chain	COL11A1	Yes [29]	cartilage condensation
P35442	Thrombospondin-2	THBS2	Yes [30]	cell adhesion
Q16363	Laminin subunit alpha-4	LAMA4	No	cell adhesion

## Data Availability

The datasets generated and/or analyzed during the current study are not publicly available due to hospital guidelines and legislation regarding personal data. Data will be available from the corresponding author on reasonable request and with permission of the Odense University Hospital Legal Department.
